# Chronic Obstructive Pulmonary Disease in Heart Failure with Reduced Ejection Fraction: Prevalence, Prognostic, and Association of Triple Inhaled Therapy

**DOI:** 10.3390/jcm15218439

**Published:** 2026-10-29

**Authors:** Nerea Aguayo-Caño, Jesus A. Torres-Zamudio, Jorge Perea-Armijo, Juan D. Martin-Diaz, Nouha Yacub-Briceño, Manuel Cabello-Terron, Enrique Enrile-Sanchez, Alvaro Roldan-Guerra, Rafael Gonzalez-Manzanares, Manuel Crespin-Crespin, Martin Ruiz-Ortiz, Dolores Mesa-Rubio, Manuel Pan-Alvarez Osorio, Manuel Anguita-Sanchez, Juan C. Castillo-Dominguez, Jose Lopez-Aguilera

**Affiliations:** 1 Heart Failure Unit, Cardiology Department, Reina Sofia University Hospital, 14004 Cordoba, Spain; nereaguayo97@gmail.com (N.A.-C.); atorzam.29@gmail.com (J.A.T.-Z.); juandiego.martindiaz@gmail.com (J.D.M.-D.); nouyacub11@gmail.com (N.Y.-B.); manuct19@gmail.com (M.C.-T.); enriqueenrilesanchez@gmail.com (E.E.-S.); alvaroroldan97@gmail.com (A.R.-G.); rafaelglezm@gmail.com (R.G.-M.); crespin2@hotmail.com (M.C.-C.); maruor@gmail.com (M.R.-O.); loladoctora@gmail.com (D.M.-R.); manuelpanalvarez@gmail.com (M.P.-A.O.); manuelanguita@secardiologia.es (M.A.-S.); juancarlos.castillo@quironsalud.es (J.C.C.-D.); mircardjla@gmail.com (J.L.-A.); 2 Cordoba Biomedical Research Institute, IMIBIC, 14004 Cordoba, Spain; 3 Center for Biomedical Research Network (CIBERCV), Carlos III Health Institute, 28029 Madrid, Spain; 4 Faculty of Health Sciences, University of Isabel I de Castilla, 09003 Burgos, Spain; 5 Medicine Faculty, University of Cordoba, 14004 Cordoba, Spain

**Keywords:** COPD, reduced LVEF, hospital admission, prognosis, mortality

## Abstract

**Background:** The coexistence of heart failure with reduced ejection fraction (HFrEF) and chronic obstructive pulmonary disease (COPD) is a common health problem that complicates therapeutic management and worsens prognosis. Our aim was to analyse the association between COPD and HF treatment optimization, long-term clinical outcomes, mortality, and HF readmissions. **Methods:** A retrospective, observational study comparing two cohorts: HFrEF with COPD versus HFrEF without COPD. Beta-blocker (BB) and ivabradine dose optimization were assessed in both groups, and inhaled treatment was assessed in patients with COPD. Clinical outcomes during follow-up were analyzed using Kaplan–Meier methods and Cox proportional hazards regression. **Results:** 375 patients were included, of whom 71 had COPD (18.9%). No significant differences were observed between groups in BB or ivabradine dose optimization. After 60 months of follow-up, patients with COPD had higher rates of all-cause mortality (40.8% vs. 25.6%; *p* = 0.014), HF-related mortality (33.8% vs. 18.4%; *p* = 0.006), and HF readmission (50.7% vs. 35.2%; *p* = 0.014). COPD remained significantly associated with adverse clinical outcomes in multivariable Cox regression analysis. Among patients with COPD, dual inhaled therapy was the most common regimen (54.9%), followed by triple inhaled therapy (32.4%). **Conclusions:** In our cohort of patients with HFrEF, concomitant COPD was associated with higher mortality and HF readmission rates despite similar BB and ivabradine dose optimization between groups. These findings highlight the importance of COPD in the clinical management of patients with HFrEF, although the observational design of the study precludes causal conclusions. Further studies are warranted to clarify the relationship between COPD, HF outcomes, and treatment patterns.

## 1. Introduction

Heart failure (HF) and chronic obstructive pulmonary disease (COPD) frequently coexist, with an estimated prevalence of 20–30% in patients with heart failure with reduced ejection fraction (HFrEF) [1,2]. However, this figure is often underestimated [3] because both conditions share dyspnoea as their main symptom, complicating differential diagnosis. Both diseases are linked by common risk factors (primarily age and smoking) and shared pathophysiological pathways, such as chronic systemic inflammation, oxidative stress, and neurohormonal activation [4,5].

The coexistence of HFrEF and COPD poses important challenges for clinical management. COPD exacerbations may contribute to cardiovascular destabilisation and increase the risk of acute cardiovascular events, including hospitalisation for HF. Furthermore, concerns regarding potential pharmacological interactions may complicate treatment decisions. Beta-blockers (BB), which are essential components of guideline-directed medical therapy for HFrEF, have historically been underused in patients with COPD because of concerns about bronchospasm [6,7,8]. However, evidence supports the use of cardioselective BB in patients with COPD when clinically indicated, particularly in those with concomitant HFrEF.

Conversely, inhaled beta-agonists may increase heart rate and potentially promote arrhythmias, raising concerns about their use in patients with underlying cardiovascular disease. These considerations highlight the importance of balancing the benefits and risks of pharmacological treatment in patients with both conditions. In this sense, recent evidence indicates that triple inhaled therapy (LAMA + LABA + inhaled corticosteroids) may improve the overall prognosis of patients with COPD by reducing cardiovascular complications [9].

Accordingly, the aims of this study are to estimate the prevalence of COPD in a cohort of patients with HFrEF and to determine its prognostic impact in terms of HF readmissions and mortality; to assess the degree of therapeutic optimisation in HFrEF, with special emphasis on the prescription and tolerance of BB; and to characterise inhaled treatment patterns in this cohort, assessing the use of triple inhaled therapy in patients with concomitant HFrEF and COPD.

## 2. Materials and Methods

### 2.1. Study Design and Population

An observational, retrospective, longitudinal, and analytical study was conducted, including all patients diagnosed with HFrEF according to the universal definition of HF [10,11] who were consecutively treated in our HF unit from January 2018 to September 2020. Patients with advanced HF who underwent heart transplantation during follow-up were excluded. Patients with HFrEF were divided into two groups, according to their clinical history of COPD: Group 1, patients with HFrEF and COPD (mixed phenotype); Group 2, patients with HFrEF without COPD.

A total of 384 patients were initially considered for inclusion. Of these, nine patients underwent heart transplantation during follow-up and were excluded from the analysis because their subsequent clinical course was no longer comparable with that of the study population. The final cohort therefore comprised 375 patients. No patients were lost to follow-up during the study period.

Clinical data, including baseline characteristics, pharmacological treatments, changes in treatment during follow-up, and clinical outcomes, were retrospectively collected from the centralised electronic health information system. This system provided access to records across the regional healthcare network, including our institution, other hospitals, emergency departments, and primary care centres. Consequently, clinical information and events recorded outside our study centre could also be retrieved. Mortality data included deaths occurring both in hospital and outside hospital settings, including deaths at home. This comprehensive data collection approach enabled longitudinal follow-up of the cohort without recorded losses to follow-up.

### 2.2. Clinical and Analytical Variables

The aetiology of HF was established considering clinical criteria and the results of complementary tests, particularly echocardiography. HF symptoms were defined according to the New York Heart Association (NYHA) functional class. Comorbidities were established based on data collected from each patient’s medical history at the start of follow-up. Specifically, the diagnosis of COPD was based on a combination of respiratory symptoms (dyspnoea, chronic cough, and wheezing), exposure to risk factors (mainly smoking), and confirmation via spirometry, usually performed by the Pulmonology Unit. The presence of an obstructive pattern (FEV1/FVC < 70%) was considered for the diagnosis, and COPD severity was defined based on the GOLD classification [12]. Hospital admission for HF was defined as any hospitalisation for HF requiring the administration of intravenous diuretics and/or inotropes.

Various baseline laboratory parameters were analysed, defining the presence of chronic kidney disease (CKD) according to the criteria established by the KDIGO guidelines [13].

The use of therapeutic measures for HF at the start of follow-up was analysed, with particular emphasis on the use of quadruple therapy (angiotensin-converting enzyme inhibitors (ACEI), angiotensin II receptor blockers (ARB-II), Angiotensin Receptor Neprilysin Inhibitor (ARNI), mineralocorticoid receptor antagonists (MRA), beta-blockers, and sodium-glucose cotransporter 2 inhibitors (SGLT2i)), loop and thiazide diuretics, or the employment of nonpharmacological measures, such as cardiac devices (implantable cardiac defibrillator (ICD) and cardiac resynchronisation therapy (CRT)) or percutaneous treatment of mitral regurgitation, using edge-to-edge repair devices.

Regarding pharmacological treatment in HFrEF, Table S1 details the classification into low, medium, and high doses for the different beta-blockers used. The ACEIs considered in our study were enalapril, lisinopril, ramipril, and captopril, whilst the ARBs included candesartan, losartan, and valsartan. However, information regarding the specific doses of ACEIs and ARBs was not available, either at baseline or at the end of follow-up.

Regarding COPD-specific management, the use of long-acting inhaled therapies to improve symptoms, reduce exacerbations, and improve quality of life was considered. These included long-acting β_2_-agonist bronchodilators (LABA: salmeterol, formoterol, and indacaterol) and long-acting anticholinergics (LAMA: tiotropium, glycopyrronium), administered as monotherapy or as part of dual or triple therapy. Dual inhaled therapy (DIT) was defined as the combination of two pharmacological agents delivered via one or two inhalation devices. Two main DIT strategies were identified according to patient requirements: (1) LABA plus LAMA (dual bronchodilation), considered the standard approach for patients who experience significant dyspnoea, providing complementary mechanisms of action on the bronchial muscle to maximise airway opening, and (2) LABA plus inhaled corticosteroid (ICS), primarily indicated in patients with overlapping asthmatic features or elevated blood eosinophil counts. Triple inhaled therapy (TIT) is the optimised step up from conventional inhaled treatment, combining three active ingredients (LAMA, LABA, and ICS), usually in a single device.

### 2.3. Echocardiographic Variables

Data from the baseline echocardiogram obtained at the start of follow-up were analysed, and relevant structural variables were collected, including left ventricular end-diastolic diameter (LVEDD, mm), left ventricular end-systolic diameter (LVESD, mm), left ventricular end-diastolic volume (LVEDV, ml), left ventricular end-systolic volume (LVESV, ml), and LVEF (%) determined by the Simpson or Teichholz method. Improvement in LVEF was defined according to the criteria established in the 2021 Universal Definition of HF [11].

### 2.4. Prognostic Variables

Patient prognosis was evaluated based on readmissions for HF, all-cause mortality, and HF-related mortality. Death from HF was defined as resulting from worsening heart failure or sudden cardiac death. Unwitnessed deaths or those of unknown cause were not considered cardiac-related in patients with life-threatening non-cardiac conditions, such as active malignancy or severe infection.

The primary endpoint was a composite of all-cause mortality or hospitalisation for HF during the follow-up period; the occurrence of either component met this endpoint. Secondary endpoints included all-cause mortality, HF-related mortality, and HF readmissions, each analysed separately.

### 2.5. Statistical Analysis

Quantitative variables were expressed as mean and standard deviation. If the distribution of values did not conform to normality, the median and interquartile range were used. Categorical variables were expressed as absolute frequencies and percentages.

The normality of quantitative variables was studied using the Shapiro–Wilk test. Variables were compared between groups using the appropriate parametric and non-parametric tests: chi-square or Fisher’s exact test for qualitative variables, and Student’s *t*-test or Mann–Whitney U test for quantitative variables. The McNemar and Wilcoxon tests were used to compare changes in qualitative and quantitative variables over time within the same group, respectively.

Event-free survival and time-to-event outcomes according to the presence of COPD were evaluated using Kaplan–Meier survival curves, with differences between groups compared using the log-rank test. These analyses were performed for the composite primary endpoint (all-cause mortality or hospitalisation for HF) as well as for all-cause mortality, HF-related mortality, and HF readmissions separately.

Univariable Cox proportional hazards models for the composite primary endpoint and for each secondary outcome (all-cause mortality, HF-related mortality, and HF readmissions) were first fitted for each candidate variable: age, sex, hypertension, diabetes mellitus, ischaemic aetiology, CKD, COPD, atrial fibrillation, anaemia, depression, obesity, embolic stroke, atherothrombotic stroke, and peripheral arterial disease. Variables with a univariable *p*-value < 0.10 were entered into the multivariable models. Results are expressed as hazard ratios (HR) with 95% confidence intervals (CI). COPD was considered an independent prognostic factor if it remained significantly associated with the outcome after adjustment (*p* < 0.05).

The discriminative ability of each multivariable model was summarised using Harrell’s concordance index (C-index), calculated in the complete-case sample. The proportional hazards assumption was evaluated using scaled Schoenfeld residuals (both global and variable-specific tests) alongside visual inspection of residual plots. The estimate for COPD was deemed robust if it remained materially unchanged.

For HF readmissions and HF-related mortality, other causes of death were treated as competing events. For these endpoints, the primary Cox models estimated cause-specific hazards, censoring patients who experienced a competing event. To assess the robustness of these estimates, sensitivity analyses were conducted using Fine–Gray subdistribution hazard models with the same covariates, expressing results as subdistribution hazard ratios (sHR) with 95% CIs, and cumulative incidence functions were compared using Gray’s test. Competing-risk methods were not applied to the composite primary endpoint or to all-cause mortality, as death is an integral component of both outcomes.

Missing data were infrequent (12 of 375 patients, 3.2%). Patients with missing data in any variable included in a given model were excluded from the corresponding multivariable analysis (complete-case analysis); multivariable models therefore included 363 patients, whereas univariable analyses included 375 patients. Baseline characteristics of patients with and without missing data were compared, and no relevant differences were observed.

A *p*-value < 0.05 was considered statistically significant (95% confidence interval). All statistical analyses were performed using IBM-SPSS software (version 25.0 for Macintosh, IBM Corp., Armonk, NY, USA) and R 4.2.1 (The R Foundation, Vienna, Austria) (https://www.R-project.org/).

## 3. Results

### 3.1. Clinical Characteristics

A total of 375 patients diagnosed with HFrEF were analysed, with a mean age of 66.8 ± 12.6 years, 76% of whom were men. Group 1 included 71 patients (18.9%), while Group 2 included the remaining 304 patients (81.1%). The baseline characteristics of both groups are shown in Table 1. Group 1 was older (70.8 ± 9.2 vs. 65.9 ± 13.2; *p* < 0.001) and showed a higher prevalence of current smoking (19.7% vs. 10.2%; *p* < 0.001) or former smokers (64.8% vs. 35.5%; *p* < 0.001) compared with the non-COPD group. There were no significant differences between the two groups in the presence of other cardiovascular risk factors, such as high blood pressure, diabetes mellitus, or dyslipidaemia. However, arteriosclerosis complications were more frequent in the COPD group, including atherothrombotic stroke (16.9% vs. 6.9%; *p* = 0.007) and the presence of peripheral artery disease (21.1% vs. 10.8%; *p* = 0.020). The prevalence of other comorbidities, such as anaemia, chronic kidney disease, or atrial fibrillation, was similar in both groups.

Regarding the peculiarities of HF, there were differential characteristics in patients with the respiratory comorbidity of COPD. These patients had a lower prevalence of de novo HF compared with the HFrEF group without COPD (42.2% vs. 56.3%, *p* = 0.033) and a lower probability of LVEF improvement during follow-up (30.9% vs. 44.1%; *p* = 0.044). Neither NYHA functional class III–IV (21.1% vs. 16.8%; *p* = 0.386) nor the rate of previous HF hospitalisations differed significantly between the groups. In terms of aetiology, ischaemic HF was the most common in both groups, with no significant differences between them (36.6% vs. 31.9%, *p* = 0.446). No differences were found in the distribution of other aetiologies of HF.

As shown in Table S2, evaluation of COPD severity among patients with HFrEF revealed a predominance of moderate spirometric impairment (GOLD 2: 57.7%), followed by GOLD 1 (22.5%). In terms of symptom and exacerbation risk groups, most patients were categorized as GOLD A (42.3%) or GOLD B (45.1%).

### 3.2. Electrocardiographic Parameters

The mean QRS duration in our cohort was 114.4 ± 29.5. A wide QRS complex was observed in 36.8% of patients, predominantly attributable to a high prevalence of left bundle branch block. No statistically significant differences were found, although there was a non-significant trend towards a higher proportion of right bundle branch block in patients with HFrEF and COPD (15.5% vs. 6.6%; *p* = 0.06) (Table 1).

### 3.3. Echocardiographic Parameters

Regarding left ventricular systolic function at baseline, no significant differences were observed between the two groups in terms of LVEF data (30.6 ± 5.6% vs. 29.8 ± 6.2%; *p* = 0.320). However, left ventricular volume was significantly higher in patients with HFrEF without COPD. In addition, a lower pulmonary artery systolic pressure was observed in patients with COPD (27.6 ± 9.1 vs. 31.8 ± 10.6, *p* = 0.018), Table 1. In terms of temporal evolution, patients with COPD were less likely to achieve LVEF improvement compared with those without COPD (30.9% vs. 44.1%; *p* = 0.044).

### 3.4. Neurohormonal Response

No significant differences were found in NT-proBNP or CA-125 levels between the two groups Table 1.

### 3.5. Treatment of Heart Failure with Reduced Ejection Fraction and Inhaled Therapy

In the overall cohort at baseline, 91.4% of patients received renin-angiotensin axis modulation therapy (ACEI, ARB, or ARNI), with 34.9% receiving ARNI. Overall, BB use reached 90.9%, and 75.2% of patients were treated with an MRA. Loop diuretic use was high (80.5%), whereas thiazides were prescribed in 19.5% of cases.

**Figure 1:**
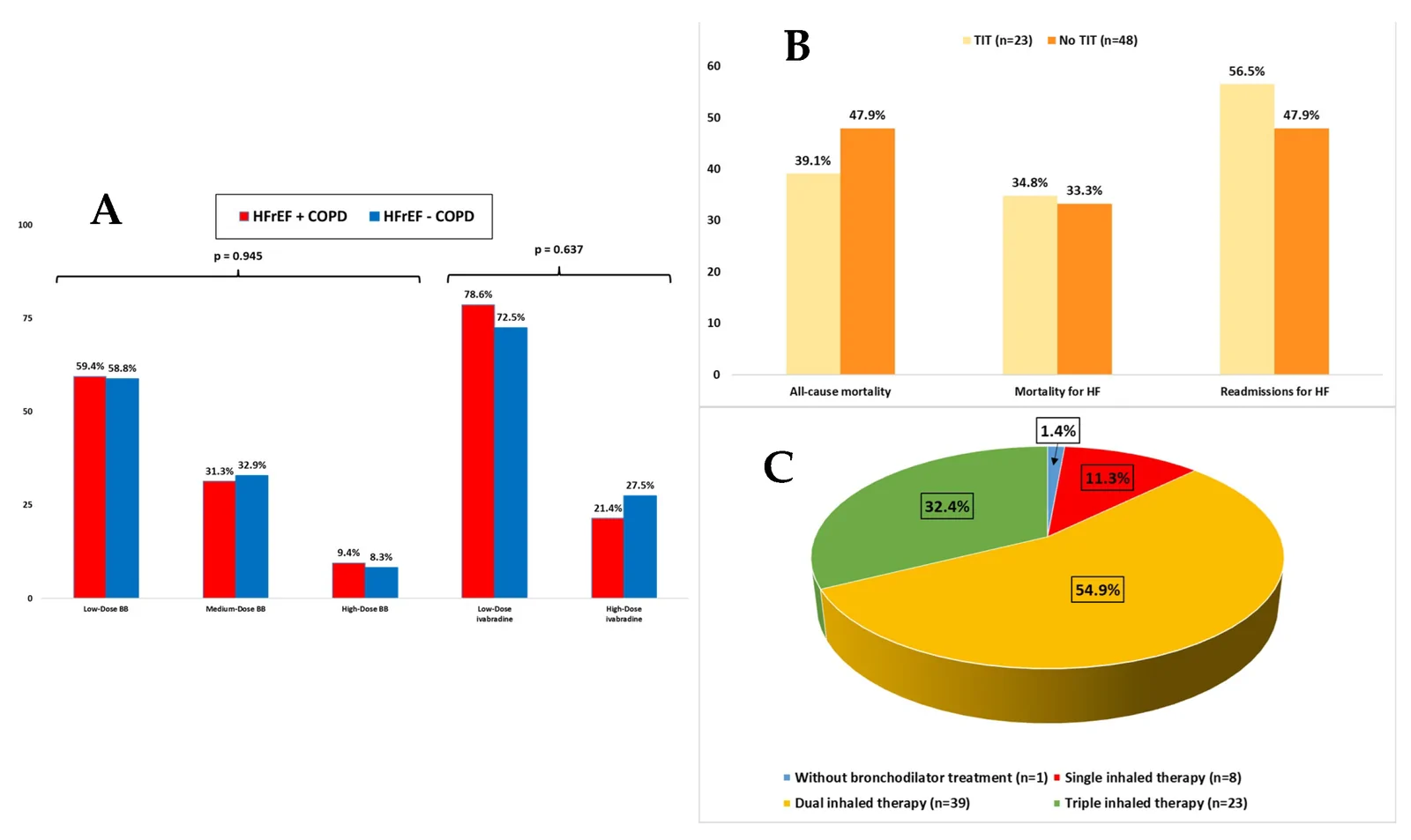
Heart failure therapy, outcomes by inhaled therapy, and bronchodilator treatment in patients with HFrEF and COPD. (**A**) Distribution of beta-blocker (BB) and ivabradine doses in patients with HFrEF and COPD (HFrEF + COPD) compared with those without COPD (HFrEF−COPD). (**B**) All-cause mortality, heart failure (HF) mortality, and hospital readmissions for HF in patients with HFrEF and COPD, according to whether they received triple inhaled therapy or not. (**C**) Inhaled bronchodilator treatment in patients with HFrEF and COPD.

When comparing groups at baseline, patients with COPD received ARNI more frequently than patients with HFrEF without COPD (46.5% vs. 32.2%; *p* = 0.023). No differences were observed in the prescription of BB, MRA, SGLT2i, diuretics, or other pharmacological agents. Regarding dosing, more than half of the patients in both groups received low doses of BB (59.4% vs. 58.8%, *p* = 0.945), and only a small percentage reached high doses in both groups (9.4% vs. 8.3%, *p* = 0.945; Table S3. No significant differences were observed in the dosage of ivabradine (Figure 1). Device therapy was limited and similar in both subgroups: ICD (12.7% in COPD vs. 9.2%; *p* = 0.378) or CRT (7.0% vs. 4.6%; *p* = 0.399).

Regarding beta-blocker therapy, prescription rates remained stable without significant differences between patients with and without COPD (90.1% vs. 91.1% at baseline; 85.9% vs. 86.5% at the end of follow-up). During follow-up, a significant upward dose titration was observed in both groups (*p* = 0.004 for COPD+ and *p* < 0.001 for COPD-), reflected by a reduction in the proportion of patients on low-dose beta-blockers and a concomitant increase in those reaching medium and high doses; Table S3. Notably, no significant differences were found in dose distribution between groups, either at baseline (*p* = 0.945) or at the end of follow-up (*p* = 0.818).

In contrast, overall medical therapy evolved similarly across cohorts during follow-up, characterized by a significant transition from ACEIs/ARBs to ARNI and an increased uptake of SGLT2 inhibitors and CRT. At the end of follow-up, pharmacological and device treatments were comparable between groups, with the sole exception of loop diuretics, which were prescribed in a significantly higher proportion of patients with COPD (85.9% vs. 67.8%; *p* = 0.002; Table S4.

With regard to inhaled therapy used in patients with HFrEF and COPD, combined inhalers represent the mainstay of bronchodilator treatment for the vast majority of patients in this cohort, with DIT (54.9%) predominating over TIT (32.4%) (Figure 1).

### 3.6. Prognosis: Readmissions for Heart Failure and Mortality

**Figure 2:**
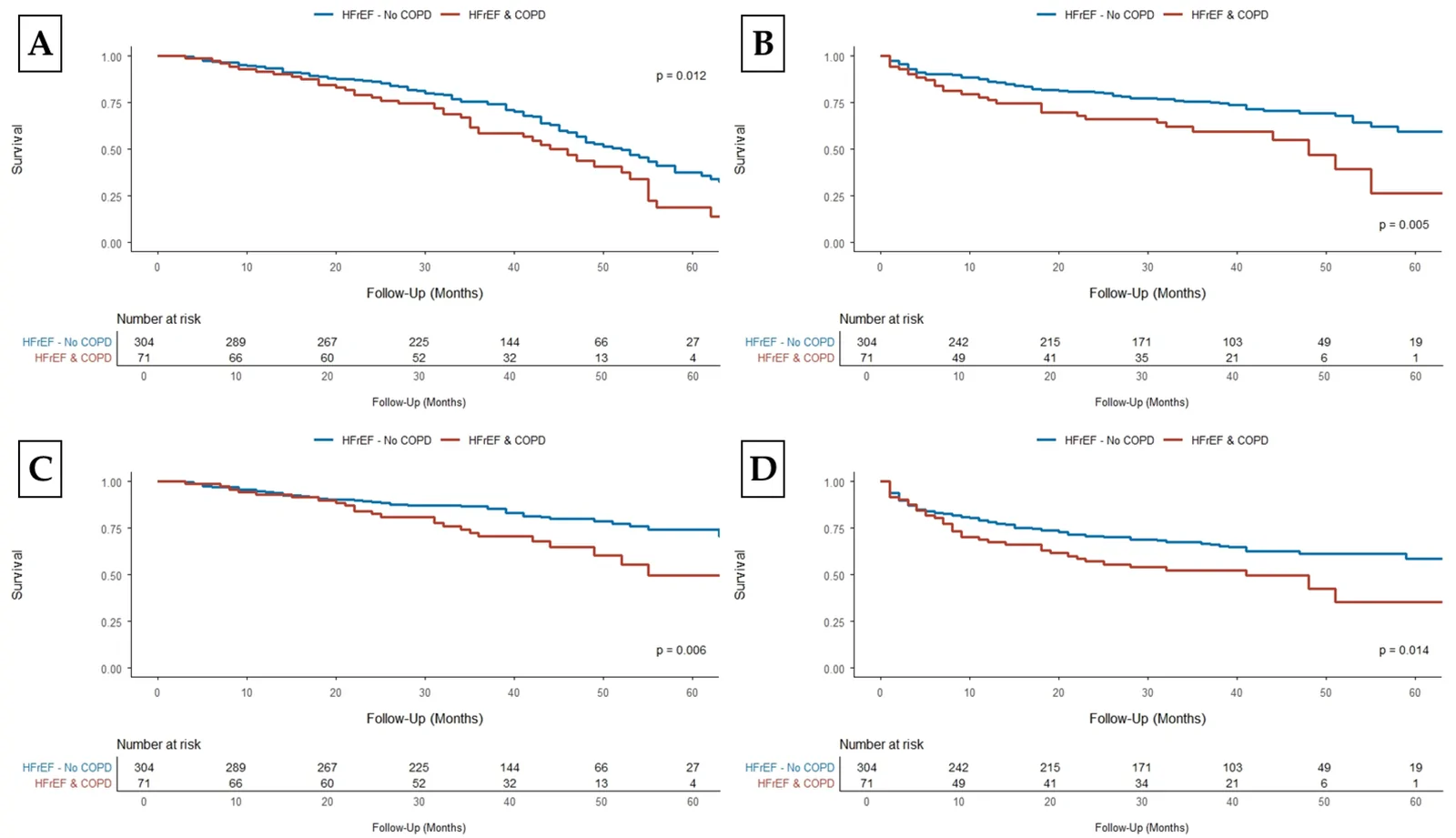
Kaplan–Meier event-free survival curves according to the presence of COPD. Kaplan–Meier analysis of outcomes in patients with HFrEF with COPD (red line) and without COPD (blue line). (**A**) Composite primary endpoint (all-cause mortality or HF hospitalisation). (**B**) All-cause mortality. (**C**) HF-related mortality. (**D**) HF readmissions. Differences between survival curves were assessed using the log-rank test. Numbers at risk at each follow-up interval are displayed below the respective panels. COPD: chronic obstructive pulmonary disease; HF: heart failure; HFrEF: heart failure with reduced ejection fraction.

The overall cohort had a median follow-up of 31 months. Patients with concomitant HFrEF and COPD exhibited significantly worse prognosis across all evaluated endpoints compared to those without COPD (Figure 2). Hospital readmissions for HF were significantly more frequent in the HFrEF group with COPD (50.7% vs. 35.2%; *p* = 0.014) (Figure S1). Furthermore, this subgroup had significantly higher mortality than patients with HFrEF without COPD, both for all-cause mortality (40.8% vs. 25.6%; *p* = 0.014) and specifically attributable to HF (33.8% vs. 18.4%; *p* = 0.006) (Figure S1). In this cohort, the use of TIT had no impact on long-term outcomes, readmissions for HF, or mortality (Figure 1).

### 3.7. Cox Regression Analysis: Predictors of the Composite Outcome and of Each Individual Outcome

In univariable Cox analysis, COPD was associated with a higher risk of the primary composite endpoint (HR 1.54, 95% CI 1.10–2.17, *p* = 0.013). This association remained significant after adjustment (adjusted HR 1.41, 95% CI 1.02–1.97, *p* = 0.048), Table 2, indicating that COPD was an independent predictor of the primary endpoint. The same pattern was observed when the components were analysed separately. In univariable analysis, COPD was associated with HF readmission (HR 1.60, 95% CI 1.09–2.33, *p* = 0.015), HF-related death (HR 1.94, 95% CI 1.20–3.13, *p* = 0.007) and all-cause mortality (HR 1.83, 95% CI 1.19–2.80, *p* = 0.006). After multivariable adjustment, COPD remained independently associated with HF readmission (adjusted HR 1.37, 95% CI 1.03–1.90, *p* = 0.044), HF-related death (adjusted HR 1.55, 95% CI 1.05–2.18, *p* = 0.042) and all-cause mortality (adjusted HR 1.49, 95% CI 1.02–2.20, *p* = 0.046). CKD and anaemia were also independently associated with all four endpoints Tables S5–S7.

The proportional hazards assumption was met for COPD in the models for the primary endpoint (*p* = 0.23), all-cause mortality (*p* = 0.57) and HF readmission (*p* = 0.19) and was borderline for HF-related death (*p* = 0.054). The hazard ratios for COPD can therefore be interpreted as average effects over the follow-up period, although the estimate for HF-related death should be interpreted with caution. Departures from proportionality in the global tests were attributable to other covariates and did not involve COPD, and the estimates for COPD were unchanged in sensitivity analyses.

In the competing-risks sensitivity analyses, 32 patients died without a prior HF readmission, and 28 patients died from causes other than HF during follow-up. In the Fine–Gray models, the independent association between COPD and clinical outcomes remained consistent with the cause-specific Cox regression estimates. Specifically, COPD remained independently associated with a higher risk of HF readmission (sHR 1.32, 95% CI 1.02–1.71, *p* = 0.038) and HF-related mortality (sHR 1.48, 95% CI 1.04–2.10, *p* = 0.029), confirming that these risks were not overestimated due to competing events. Chronic kidney disease and anaemia also remained independently associated with both endpoints, whereas obesity maintained its inverse relationship with HF readmissions. In unadjusted cumulative incidence analyses (Gray’s test), patients with COPD exhibited a significantly higher cumulative incidence of HF readmission (*p* = 0.039) and HF-related death (*p* = 0.005), with no significant differences between groups regarding the cumulative incidence of competing events (*p* = 0.33 and *p* = 0.97, respectively). Overall, these sensitivity analyses suggest that COPD is a robust and independent prognostic factor in HFrEF and that its predictive value is not confounded or biased by competing risks.

## 4. Discussion

The coexistence of COPD and HFrEF represents a clinically challenging overlap that complicates diagnosis, treatment optimization, and long-term care. The findings of our real-world study, with a five-year follow-up, not only confirm the high prevalence of this association but also demonstrate its adverse prognostic impact. Across Kaplan–Meier and multivariable Cox proportional hazards analyses, COPD was independently associated with a higher incidence of the composite primary endpoint (all-cause mortality or HF hospitalisation). Furthermore, this prognostic detriment was consistent across each individual component, including all-cause mortality, HF-related death, and HF readmissions. Importantly, these findings remained robust after accounting for competing risks using Fine–Gray subdistribution models.

### 4.1. Prevalence and Clinical Characteristics

The prevalence of COPD in our HFrEF population is similar to previously reported data. Notably, our cohort showed a clear predominance of moderate airflow obstruction (GOLD 2), alongside a low incidence of previous COPD exacerbations, with the vast majority of patients categorized into low-risk groups (GOLD A and B). However, as reported by Kubota et al. and Leong et al., the true prevalence is likely underestimated due to symptom overlap and diagnostic ambiguity [6,14]. Dyspnoea, a cardinal symptom in both conditions, often leads to a detection bias where the clinician prioritises one disease over another, hindering early and accurate diagnosis. As previous studies have pointed out, overlapping symptoms often lead to misinterpretation of decompensations [2], so that up to 20% of patients admitted for an apparent respiratory exacerbation actually have cardiac decompensation as the main or contributing cause. This diagnostic confusion may delay the initiation of optimal diuretic treatment and haemodynamic stabilisation, thereby increasing the likelihood of readmissions for HF.

Regarding the clinical characteristics, in our cohort, the overrepresentation of male patients and the heightened prevalence of smoking, particularly within the COPD subgroup, may partly reflect the historically higher smoking exposure among men in these age brackets. Since cumulative tobacco exposure is intrinsically associated with COPD, this pattern may have contributed to the increased prevalence of the disease in male subjects. However, the observational design of our study does not allow us to establish a causal relationship between sex, smoking exposure, and COPD prevalence.

### 4.2. Optimisation of Treatment for Heart Failure with Reduced Ejection Fraction

A key and notable finding of our study is the suboptimal titration of BB in terms of dose. Despite robust evidence supporting their prognostic benefit in HFrEF [15], almost half of our patients were still taking low doses at the end of the follow-up period, a phenomenon also observed in large international registries of patients with HFrEF and COPD, such as ASIAN-HF [6]. Historically, the use of BB in patients with respiratory disease has been limited by concerns regarding potential bronchospasm [6]. However, our real-world data indicate that the presence of COPD was not the main determining factor for underuse, since prescription rates and dose distribution were almost identical in patients with and without COPD. This finding suggests the notion of a broader “clinical inertia” that transcends respiratory comorbidity, similarly described in the Japanese cohort of the ASIAN-HF registry [6] and the Colombian RECOLFACA registry [16]. It is therefore essential to overcome the theoretical concerns of bronchial adverse effects associated with the use of cardioselective BB [2,6]. Robust evidence supports their safety and survival benefit in this population [2,6], and even their withdrawal during an exacerbation increases the risk of death. Likewise, the limited use of ivabradine represents a missed opportunity to achieve heart rate control without compromising pulmonary function, a strategy endorsed by current clinical guidelines [17].

During follow-up, disease-modifying therapy for HFrEF was successfully prescribed across the overall cohort, with no significant differences observed in the prescription or dosing of key prognostic drugs between patients with and without COPD. The sole exception was loop diuretics, which were prescribed in a significantly higher proportion of COPD patients by the end of follow-up. This was because, in patients without COPD, there was a decrease in prescriptions. This may be linked to the higher rate of readmissions for heart failure observed in the COPD group during the follow-up period, which highlights their greater susceptibility to fluid overload and the inability to discontinue or reduce diuretic treatment due to the need to maintain fluid-depleting therapy in these patients.

The advent of quadruple therapy has significantly changed the paradigm of HFrEF treatment. In our cohort, the SGLT2i usage was consistent with findings from the DAPA-HF subanalysis [18], confirming that their benefits are maintained regardless of COPD status. Given their neutral respiratory profile, these agents provide a valuable strategy when BB titration is limited. Furthermore, emerging evidence regarding mineralocorticoid receptor blockade, including recent data on finerenone from the FINEARTS-HF trial [19], suggests that non-steroidal mineralocorticoid receptor antagonists are both safe and effective in this clinical profile. As COPD is associated with systemic inflammation and pulmonary fibrosis, mineralocorticoid receptor blockade may have potential pleiotropic effects beyond its haemodynamic benefits [20]. Consequently, neurohormonal modulation with BB, renin–angiotensin–aldosterone system blockers and SGLT2i should not be withheld in patients with HFrEF, regardless of COPD status [21].

### 4.3. Inhaled Therapy

The analysis of respiratory therapy in our cohort reflects an advanced management strategy, with one-third of patients receiving triple inhaled therapy (LAMA/LABA/ICS) and more than half receiving dual therapy, despite a predominance of moderate obstruction and few previous exacerbations. The importance of this treatment lies not only in the improvement of lung function but also in its indirect cardiovascular impact. COPD exacerbations represent major triggers for HF decompensation through mechanisms involving sympathetic activation, systemic stress, and hypoxia [21], and their prevention appears to have an impact on reducing HF admissions and mortality [9]. Therefore, the optimisation of inhaled therapy should be considered an integral component of the neurohormonal management of HF, even though these benefits were not observed in terms of hard endpoints in our series.

Our results reinforce the need for a multidisciplinary approach. Table 1 reveals that COPD patients showed a higher prevalence of active smoking, as well as a greater tendency towards chronic kidney disease, which further complicates therapeutic titration [18,19,22]. Integrated management of these comorbidities is essential to implement aggressive BB titration strategies and ensure that inhaled therapy, combined with smoking cessation, can minimise the exacerbations that precipitate HF admissions. The role of nursing is also essential in this multidisciplinary approach [21,23]. Published data show that, at the time of discharge, patients are 21% less likely to be referred for cardiology follow-up and 6% less likely to be referred to multidisciplinary HF teams [21,24], highlighting a significant gap in healthcare.

### 4.4. Prognosis

Another key finding of our study is the marked and persistent divergence in long-term outcomes between groups. Multivariable Cox regression analysis confirmed that COPD in patients with HFrEF is an independent risk factor associated with the composite endpoint of all-cause mortality and heart failure readmission. Notably, this independent association remained statistically significant for each individual component when evaluated separately, including heart failure readmissions, heart failure mortality, and all-cause mortality. Although analysis of baseline characteristics shows that the COPD group had a significantly higher mean age [6], the discrepancy in HF-specific mortality suggests that respiratory disease acts as an independent factor in the progression of HF, regardless of ageing [25]. The five-year Kaplan–Meier curves reflect an early and sustained decline in the COPD group, highlighting the prognostic vulnerability inherent in this patient phenotype in routine clinical practice [26].

Pathophysiologically, this deleterious interaction is driven by a shared systemic inflammatory state. COPD promotes the release of proinflammatory cytokines that could accelerate adverse remodelling and myocardial fibrosis. Additionally, chronic hypoxaemia and alterations in intrathoracic pressure, secondary to airflow limitation, increase right ventricular afterload. This haemodynamic burden precipitates episodes of left and right heart failure, which are often more difficult to manage clinically. Furthermore, chronic hypoxaemia may contribute to chemoreflex and autonomic dysregulation, providing an additional pathophysiological link between COPD and HF. Persistent hypoxaemia can increase peripheral chemoreceptor activation, leading to enhanced sympathetic outflow and impaired autonomic balance. This sustained sympathetic overactivity may increase heart rate, vascular tone and myocardial oxygen demand, while also promoting adverse ventricular remodelling and arrhythmogenic susceptibility. Recent evidence has highlighted the role of altered chemoreflex function and sympathetic activation in COPD and their potential contribution to cardiovascular disease, including heart failure [27]. Therefore, chemoreflex-mediated autonomic dysfunction may represent an additional mechanism through which chronic respiratory abnormalities contribute to the progression and poor prognosis of HF in patients with COPD.

In our real-world cohort, with a five-year follow-up period, patients with concomitant COPD exhibited significantly higher rates of HF-related admissions compared with those without respiratory comorbidity, further supporting this pathophysiological interplay [22]. Notably, long-term real-world studies evaluating this association remain scarce in the current literature.

### 4.5. Limitations

We acknowledge several limitations in this study. First, this was a single-centre observational study, which limits the generalisability of our findings and precludes establishing direct causal relationships. Second, the relatively small sample size of patients with COPD, particularly regarding subgroup analyses across different inhaled treatment regimens, may have limited the statistical power to detect smaller differences. Third, residual confounding cannot be entirely excluded, and potential misclassification of COPD remains possible given the observational nature of the design and the heterogeneity of clinical presentations. Furthermore, treatment selection may have been influenced by clinical factors not fully accounted for in the analysis, such as COPD severity, exacerbation history, or blood eosinophil counts.

Fourth, information regarding baseline and follow-up doses of ACE inhibitors and ARBs was not systematically recorded, preventing the evaluation of dose optimization and treatment intensity over time. Fifth, the relatively low prescription rate of SGLT2 inhibitors in both cohorts reflects the practice standards during the inclusion period, which may limit the direct extrapolation of our results to contemporary therapeutic settings. Nevertheless, the statistically significant differences observed in hard clinical endpoints—including mortality and HF readmissions—together with the consistency of the longitudinal follow-up lend meaningful clinical validity to our findings.

## 5. Conclusions

In conclusion, COPD appears to be an independent predictor of adverse outcomes in patients with HFrEF, showing a significant association with the primary composite endpoint (all-cause mortality or HF readmission) and each of its individual components. These observations reinforce the need for multidisciplinary care and optimized medical therapy, including beta-blocker titration and bronchodilator use. However, given the observational design and inherent limitations of this study, these results should be interpreted with caution and warrant confirmation in larger, prospective future studies.

## Figures and Tables

**Table 1:** Baseline clinical characteristics.

	HFrEF (N = 375)	HFrEF COPD (+) (N = 71)	HFrEF COPD (−) (N = 304)	*p* Value
Age (years)	66.8 ± 12.6	70.8 ± 9.2	65.9 ± 13.2	<0.001
Sex male	284 (75.7%)	67 (94.4%)	217 (71.4%)	<0.001
Hypertension	249 (66.4%)	51 (71.8%)	198 (65.1%)	0.282
Diabetes mellitus	179 (47.7%)	36 (50.7%)	143 (47.0%)	0.578
Dyslipidaemia	237 (63.2%)	45 (63.3%)	192 (63.1%)	0.998
Smoking				
Current smoker	45 (12.0%)	14 (19.7%)	31 (10.2%)	<0.001
Former smoker	154 (41.1%)	46 (64.8%)	108 (35.5%)	
De novo heart failure	201 (53.6%)	30 (42.2%)	171 (56.3%)	0.033
Hospital admission for heart failure				
0–1	330 (88.0%)	59 (83.1%)	271 (89.1%)	0.097
≥2	42 (11.2%)	12 (16.9%)	30 (9.8%)	
Heart failure duration (months)	37.5 ± 66.5	49.7 ± 73.6	34.7 ± 68.5	0.091
Time to left ventricular ejection fraction improvement (months)	16.3 ± 7.7	16.4 ± 8.9	16.3 ± 9.8	0.874
NYHA				
I–II	309 (82.4%)	56 (78.9%)	253 (83.2%)	0.386
III–IV	66 (17.6%)	15 (21.1%)	51 (16.8%)	
Aetiology				
Ischaemic	123 (32.8%)	26 (36.6%)	97 (31.9%)	0.446
Valvular	6 (1.6%)	0 (0%)	6 (2%)	0.233
Tachycardiomyopathy	39 (10.4%)	5 (7.0%)	34 (11.2%)	0.303
Oncological	15 (4%)	1 (1.4%)	14 (4.6%)	0.216
Idiopathic	113 (30.1%)	25 (35.2%)	88 (28.9%)	0.300
Atrial fibrillation	195 (52.0%)	41 (57.7%)	154 (50.6%)	0.282
Atherothrombotic stroke	33 (8.8%)	12 (16.9%)	21 (6.9%)	0.007
Embolic stroke	19 (5.1%)	1 (1.4%)	18 (5.9%)	0.119
Peripheral arterial disease	48 (12.8%)	15 (21.1%)	33 (10.8%)	0.020
Depression	53 (14.1%)	11 (15.5%)	42 (13.8%)	0.715
BMI >30	140 (37.3%)	33 (46.5%)	107 (35.2%)	0.137
QRS (ms)	114.4 ± 29.5	117.3 ± 28.5	113.7 ± 29.8	0.491
Wide QRS (%)	138 (36.8%)	31 (43.7%)	107 (35.2%)	0.360
RBBB (%)	31 (8.3%)	11 (15.5%)	20 (6.6%)	0.060
LBBB (%)	105 (28.0%)	19 (26.8%)	86 (28.3%)	
LVEF (%)	29.9 ± 6.1	30.6 ± 5.6	29.8 ± 6.2	0.320
LVEDD (mm)	62.9 ± 8.4	62.5 ± 6.9	63.0 ± 8.8	0.691
LVESD (mm)	53.3 ± 9.0	50.9 ± 8.1	53.9 ± 9.2	0.091
LVEDV (mL)	165.1 ± 61.7	152.6 ± 47.3	168.4 ± 64.7	0.047
LVESV (mL)	115.3 ± 47.8	104.7 ± 35.6	118.2 ± 50.3	0.028
TAPSE (mm)	18.2 ± 4.2	18.7 ± 4.5	18.1 ± 4.2	0.047
PASP (mm Hg)	31.0 ± 10.4	27.6 ± 9.1	31.8 ± 10.6	0.018
CKD	155 (41.3%)	36 (50.7%)	119 (39.1%)	0.075
IIIa	50 (13.3%)	11 (15.5%)	39 (12.8%)	0.440
IIIb	61 (16.0%)	15 (21.1%)	46 (15.1%)	
IV	30 (8.0%)	6 (8.5%)	24 (7.9%)	
V	14 (3.7%)	4 (5.6%)	10 (3.3%)	
Creatinine (mg/dL)	1.2 ± 0.7	1.2 ± 0.5	1.2 ± 0.8	0.988
eGFR (mL/min)	66.6 ± 26.8	65.4 ± 26.5	66.9 ± 26.8	0.669
Anaemia	116 (30.9%)	26 (36.6%)	90 (29.6%)	0.250
Hb (g/dL)	13.7 ± 2.1	13.9 ± 1.9	13.6 ± 2.21	0.256
Iron (µg/dL)	68.9 ± 57.8	59.9 ± 30.7	71.3 ± 62.8	0.236
Ferritin (µg/L)	230.6 ± 420.9	258.3 ± 533.7	223.9 ± 389.8	0.552
Transferrin (mg/L)	255.2 ± 55.7	241.7 ± 54.1	258.6 ± 55.6	0.027
TSAT (%)	20.7 ± 13.8	20.3 ± 9.5	20.8 ± 14.3	0.808
CA-125 (U/L)	61.4 ± 107.9	57.1 ± 79.9	62.4 ± 114.8	0.825
NT-proBNP (pg/mL)	10,097.2 ± 23,302.6	8697.6 ± 9364.4	10,448.9 ± 25,614.6	0.761
HbA1c (%)	6.6 ± 1.4	6.5 ± 1.0	6.7 ± 1.4	0.324
Treatment				
ACEI/ARB	212 (56.5%)	34 (47.9%)	178 (58.5%)	0.103
ARNI	131 (34.9%)	33 (46.5%)	98 (32.2%)	0.023
BB	341 (90.9%)	64 (90.1%)	277 (91.1%)	0.796
Loop diuretics	302 (80.5%)	60 (84.5%)	242 (79.6%)	0.348
Thiazides	73 (19.5%)	14 (19.5%)	59 (19.4%)	0.953
MRA	282 (75.2%)	52 (73.2%)	230 (75.6%)	0.671
Ivabradine	83 (22.1%)	14 (19.7%)	69 (22.7%)	0.586
Digoxin	44 (11.7%)	8 (11.3%)	36 (11.8%)	0.892
SGLT2i	67 (17.8%)	11 (15.5%)	56 (18.4%)	0.562
ICD	37 (9.8%)	9 (12.7%)	28 (9.2%)	0.378
CRT	19 (5.1%)	5 (7.0%)	14 (4.6%)	0.399
PMVR	1 (0.3%)	0 (0%)	1 (0.3%)	0.628
Haemodynamic				
Heart rate (bpm)	72.0 ± 13.8	71.7 ± 12.3	72.1 ± 14.1	0.842
SBP (mmHg)	111.9 ± 18.9	110.7 ± 15.9	112.2 ± 18.8	0.578
DBP (mmHg)	69.0 ± 11.3	67.7 ± 11.1	69.3 ± 11.3	0.310

Abbreviations: ACEI: angiotensin-converting enzyme inhibitors; ARB: angiotensin II receptor blockers; ARNI: angiotensin receptor–neprilysin inhibitors; BB: Beta-blocker; BMI: body mass index; CKD: chronic kidney disease; COPD: chronic obstructive pulmonary disease; CRT: cardiac resynchronization therapy; DBP: diastolic blood pressure; eGFR: estimated glomerular filtration rate; Hb: hemoglobin; HbA1c: glycated hemoglobin; HFrEF: heart failure with reduced ejection fraction; ICD: implantable cardioverter defibrillator; LVEDD: left ventricular end-diastolic diameter; LVEDV: left ventricular end-diastolic volume; LVEF: left ventricular ejection fraction; LVESD: left ventricular end-systolic diameter; LVESV: left ventricular end-systolic volume; MRA: mineralocorticoid receptor antagonists; NT-proBNP: N-terminal pro-brain natriuretic peptide; NYHA: New York Heart Association classification; PASP: pulmonary artery systolic pressure; PMVR: percutaneous mitral valve repair; SBP: systolic blood pressure; SGLT2i: sodium–glucose cotransporter 2 inhibitors; TSAT: transferrin saturation.

**Table 2:** Multivariable Cox regression analysis of independent predictors associated with the primary composite endpoint (heart failure readmission or all-cause mortality).

	Univariate Analysis			Multivariate Analysis		
	HR	95% CI	*p*-Value	HR	95% CI	*p*-Value
Age (per year)	1.04	1.02–1.05	<0.001	1.00	0.99–1.02	0.750
Sex male	1.21	0.86–1.70	0.270			
HTN	1.76	1.24–2.50	0.002	1.40	0.95–2.07	0.050
DM	1.19	0.89–1.60	0.240			
Ischaemic aetiology	1.31	0.97–1.78	0.080	0.87	0.62–1.22	0.410
COPD	1.54	1.10–2.17	0.013	1.41	1.02–1.97	0.048
CKD	3.72	2.73–5.09	<0.001	2.21	1.52–3.21	<0.001
AF	1.43	1.06–1.93	0.018	1.45	1.04–2.01	0.048
Anaemia	3.52	2.61–4.74	<0.001	2.21	1.54–3.19	<0.001
Depression	1.24	0.83–1.86	0.290			
Obesity	0.83	0.69–0.99	0.039	0.85	0.69–1.03	0.097
Embolic stroke	0.85	0.46–1.58	0.610			
Atherothrombotic stroke	1.46	0.93–2.29	0.099	1.32	0.82–2.14	0.260
Peripheral arterial disease	1.75	1.19–2.56	0.004	1.09	0.71–1.68	0.680

Abbreviations: AF: Atrial fibrillation; CI: Confidence interval; CKD: Chronic kidney disease; COPD: Chronic obstructive pulmonary disease; DM: Diabetes mellitus; HTN: Hypertension; HR: Hazard ratio.

## Data Availability

No material from other sources was reproduced in this manuscript. All content is original. The data presented in this study are available on request from the corresponding author. The data are not publicly available due to privacy and ethical restrictions.

## Supplementary Materials

The following supporting information can be downloaded at https://www.mdpi.com/article/10.3390/jcm15218439/s1. Table S1. Pharmacological dosage stratification. Table S2. Distribution of COPD severity stage in patients with HFrEF and COPD. Table S3. Beta-blocker dose distribution at baseline and end of follow-up according to COPD status. Table S4. Pharmacological and device therapies at baseline and end of follow-up in patients with HFrEF with and without COPD. Table S5. Multivariable Cox regression analysis of independent factors associated with readmissions for heart failure. Table S6. Multivariable Cox regression analysis of independent factors associated with mortality for heart failure. Table S7. Multivariable Cox regression analysis of independent factors associated with all-cause mortality. Figure S1. Proportions of clinical endpoints according to the presence of COPD.

## Abbreviations

The following abbreviations are used in this manuscript:

ACEI	Angiotensin-converting enzyme inhibitors
AF	Atrial fibrillation
ARB	Angiotensin II receptor blockers
ARNI	Angiotensin receptor neprilysin inhibitor
BB	Beta-blockers.
BMI	Body mass index
CA-125	Carbohydrate antigen 125
CI	Confidence interval
CKD	Chronic kidney disease
COPD	Chronic obstructive pulmonary disease.
CRT	Cardiac resynchronisation therapy
DBP	Diastolic blood pressure
DIT	Dual inhaled therapy
DM	Diabetes mellitus
eGFR	Estimated glomerular filtration rate
FEV1	Forced expiratory volume in 1 s
FVC	Forced vital capacity
GOLD	Global Initiative for Chronic Obstructive Lung Disease
HF	Heart failure
HFrEF	Heart failure with reduced ejection fraction
HR	Hazard ratio
HTN	Hypertension
ICD	Implantable cardiac defibrillator
ICS	Inhaled corticosteroids
KDIGO	Kidney Disease: Improving Global Outcomes
LABA	Long-acting beta2-agonist
LAMA	Long-acting muscarinic antagonist
LBBB	Left bundle branch block
LVEDD	Left ventricular end-diastolic diameter
LVEDV	Left ventricular end-diastolic volume
LVEF	Left ventricular ejection fraction
LVESD	Left ventricular end-systolic diameter
LVESV	Left ventricular end-systolic volume
MRA	Mineralocorticoid receptor antagonists
NT-proBNP	N-terminal pro-brain natriuretic peptide
NYHA	New York Heart Association
PASP	Pulmonary artery systolic pressure
PMVR	Percutaneous mitral valve repair
RBBB	Right bundle branch block
SBP	Systolic blood pressure
SGLT2i	Sodium-glucose cotransporter 2 inhibitors.
TAPSE	Tricuspid annular plane systolic excursion
TIT	Triple inhaled therapy
TSAT	Transferrin saturation
