# Supplementary material for: Chronic Obstructive Pulmonary Disease in Heart Failure with Reduced Ejection Fraction: Prevalence, Prognostic, and Association of Triple Inhaled Therapy
Source: J Clin Med. 2026 Oct 29;15(21):8439. doi: 10.3390/jcm15218439 (PMC13764631; doi:10.3390/jcm15218439)
Supplement: Supplementary file 1 [file jcm-15-08439-s001.zip › jcm-4590491-supplementary.pdf]

SUPPLEMENTARY MATERIAL

Table S1. Pharmacological dosage stratification.

Pharmacological Group	Low Dose	Medium Dose	High Dose
Beta-blockers			
Bisoprolol	< 2.5 mg QD	2.5-5 mg QD	> 5 mg QD
Carvedilol	< 12.5 mg BID	12.5-25 mg BID	≥ 25 mg BID
Nebivolol	≤ 2.5 mg QD	2.5-5 mg QD	> 5 mg QD
Atenolol*	< 50 mg QD	50-75 mg QD	> 75 mg QD
Ivabradine	5 mg BID		7.5 mg BID

**Atenolol is not currently recommended as a first-line disease-modifying therapy for HFrEF but was included due to its prescription in a minority of the cohort.*
Abbreviations: QD: Quaque die. BID: Bis in die.

Table S2. Distribution of COPD severity stage in patients with HFrEF and COPD.

	**HFrEF COPD (+) (N = 71)**
**GOLD 1**	16 (22.5%)
**GOLD 2**	41 (57.7%)
**GOLD 3**	12 (16.9%)
**GOLD 4**	2 (2.8%)
**GOLD A**	30 (42.3%)
**GOLD B**	32 (45.1%)
**GOLD E**	9 (12.7%)

Abbreviations: COPD: chronic obstructive pulmonary disease; GOLD: Global Initiative for Chronic Obstructive Lung Disease; HFrEF: heart failure with reduced ejection fraction.

Table S3. Beta-blocker dose distribution at baseline and end of follow-up according to COPD status.

	HFrEF COPD (+) (N = 71)			HFrEF COPD (-) (N = 304)		
	**Baseline**	**End**	**P value**	**Baseline**	**End**	**P value**
**Low dose**	38 (59.4%)	28 (45.9%)	0.004	163 (58.8%)	130 (49.4%)	<0.001
**Medium dose**	20 (31.3%)	23 (37.7%)		91 (32.9%)	88 (33.5%)	
**High dose**	6 (9.4%)	10 (16.4%)		23 (8.3%)	45 (17.1%)	

COPD: chronic obstructive pulmonary disease; HFrEF: heart failure with reduced ejection fraction.

Table S4. Pharmacological and device therapies at baseline and end of follow-up in patients with HFrEF with and without COPD.

	HFrEF COPD (+) (N = 71)			HFrEF COPD (-) (N = 304)		
	**Baseline**	**End**	**P value**	**Baseline**	**End**	**P value**
**ACEI/ARB**	34 (47.9%)	21 (29.6%)	0.006	178 (58.5%)	84 (27.6%)	<0.001
**ARNI**	33 (46.5%)	44 (62%)	0.027	98 (32.2%)	190 (62.5%)	<0.001
**BB**	64 (90.1%)	61 (85.9%)	0.321	277 (91.1%)	263 (86.5%)	0.013
**Loop diuretics**	60 (84.5%)	61 (85.9%)	0.810	242 (79.6%)	206 (67.8%)	<0.001
**MRA**	52 (73.2%)	48 (67.6%)	0.321	230 (75.6%)	222 (73%)	0.326
**Ivabradine**	14 (19.7%)	13 (18.3%)	0.741	69 (22.7%)	62 (20.4%)	0.250
**SGLT2i**	11 (15.5%)	25 (35.2%)	0.001	56 (18.4%)	118 (38.8%)	<0.001
**ICD**	9 (12.7%)	12 (16.9%)	0.083	28 (9.2%)	61 (20.1%)	<0.001
**CRT**	5 (7.0%)	13 (18.3%)	0.004	14 (4.6%)	55 (18.1%)	<0.001
**PMVR**	0 (0%)	1 (1.4%)	0.321	1 (0.3%)	14 (4.6%)	<0.001

ACEI: angiotensin-converting enzyme inhibitor; AF: atrial fibrillation; ARB: angiotensin II receptor blocker; ARNI: angiotensin receptor–neprilysin inhibitor; BB: beta-blocker; COPD: chronic obstructive pulmonary disease; CRT: cardiac resynchronisation therapy; HFrEF: heart failure with reduced ejection fraction; ICD: implantable cardioverter-defibrillator; MRA: mineralocorticoid receptor antagonist; PMVR: percutaneous mitral valve repair; SGLT2i: sodium-glucose cotransporter-2 inhibitor.

Table S5. Multivariable Cox regression analysis of independent factors associated with readmissions for heart failure.

	UNIVARIATE ANALYSIS			MULTIVARIATE ANALYSIS		
	HR	95% CI	p-value	HR	95% CI	p-value
Age (per year)	1.04	1.02-1.05	<0.001	1.00	0.98-1.02	0.890
Sex male	1.26	0.87-1.82	0.220			
HTN	1.96	1.33-2.89	<0.001	1.36	0.89-2.07	0.160
DM	1.06	0.77-1.47	0.720			
Ischaemic aetiology	1.62	1.16-2.26	0.005	1.11	0.77-1.60	0.570
COPD	1.60	1.09-2.33	0.015	1.37	1.03-1.90	0.044
CKD	4.00	2.82-5.68	<0.001	2.25	1.48-3.43	<0.001
AF	1.53	1.09-2.14	0.014	1.48	1.03-2.13	0.076
Anaemia	3.77	2.71-5.25	<0.001	2.16	1.46-3.19	<0.001
Depression	1.09	0.68-1.73	0.720			
Obesity	0.74	0.60-0.91	0.005	0.73	0.59-0.91	0.006
Embolic stroke	1.32	0.67-2.60	0.420			
Atherothrombotic stroke	1.81	1.11-2.93	0.017	1.68	1.01.-2.81	0.047
Peripheral arterial disease	1.73	1.12-2.67	0.013	0.99	0.62-1.59	0.970

Abbreviations: AF: Atrial fibrillation; CI: Confidence interval; CKD: Chronic kidney disease; COPD: Chronic obstructive pulmonary disease; DM: Diabetes mellitus; HTN: Hypertension; HR: Hazard ratio.

Table S6. Multivariable Cox regression analysis of independent factors associated with mortality for heart failure.

	UNIVARIATE ANALYSIS			MULTIVARIATE ANALYSIS		
	HR	95% CI	p-value	HR	95% CI	p-value
**Age (per year)**	1.05	1.03-1.08	<0.001	1.03	1.00-1.05	0.018
**Sex male**	0.88	0.51-1.50	0.630			
**HTN**	1.72	1.03-2.88	0.039	0.99	0.58-1.70	0.980
**DM**	1.06	0.68-1.64	0.800			
**Ischaemic aetiology**	1.91	1.23-2.96	0.004	1.13	0.71-1.81	0.600
**COPD**	1.94	1.20-3.13	0.007	1.55	1.05-2.18	0.042
**CKD**	5.19	3.15-8.55	<0.001	2.54	1.43-4.52	0.001
**AF**	1.41	0.90-2.20	0.130			
**Anaemia**	4.82	3.06-7.59	<0.001	2.31	1.37-3.89	0.002
**Depression**	1.01	0.53-1.91	0.980			
**Obesity**	0.80	0.60-1.05	0.110			
**Embolic stroke**	1.35	0.58-3.12	0.480			
**Atherothrombotic stroke**	1.38	0.69-2.77	0.360			
**Peripheral arterial disease**	2.62	1.58-4.34	<0.001	1.67	0.96-2.90	0.069

Abbreviations: AF: Atrial fibrillation; CI: Confidence interval; CKD: Chronic kidney disease; COPD: Chronic obstructive pulmonary disease; DM: Diabetes mellitus; HTN: Hypertension; HR: Hazard ratio.

Table S7. Multivariable Cox regression analysis of independent factors associated with all-cause mortality.

	UNIVARIATE ANALYSIS			MULTIVARIATE ANALYSIS		
	**HR**	**95% CI**	**p-value**	**HR**	**95% CI**	**p-value**
**Age (per year)**	1.05	1.04-1.07	<0.001	1.01	0.99-1.03	0.290
**Sex male**	0.90	0.56-1.43	0.640			
**HTN**	2.09	1.33-3.29	0.001	1.56	0.94-2.58	0.087
**DM**	1.29	0.88-1.88	0.190			
**Ischaemic aetiology**	2.00	1.37-2.93	<0.001	1.06	0.70-1.59	0.790
**COPD**	1.83	1.19-2.80	0.006	1.49	1.02-2.20	0.046
**CKD**	5.68	3.72-8.69	<0.001	2.30	1.37-3.85	0.002
**AF**	1.28	0.87-1.87	0.210			
**Anaemia**	6.07	4.09-9.01	<0.001	3.39	2.09-5.48	<0.001
**Depression**	1.27	0.77-2.12	0.350			
**Obesity**	0.73	0.57-0.92	0.009	0.76	0.58-0.99	0.042
**Embolic stroke**	1.20	0.55-2.59	0.650			
**Atherothrombotic stroke**	1.62	0.89-2.97	0.120			
**Peripheral arterial disease**	2.58	1.65-4.05	<0.001	1.43	0.87-2.35	0.160

Abbreviations: AF: Atrial fibrillation; CI: Confidence interval; CKD: Chronic kidney disease; COPD: Chronic obstructive pulmonary disease; DM: Diabetes mellitus; HTN: Hypertension; HR: Hazard ratio.

Figure S1. Proportions of clinical endpoints according to the presence of COPD.

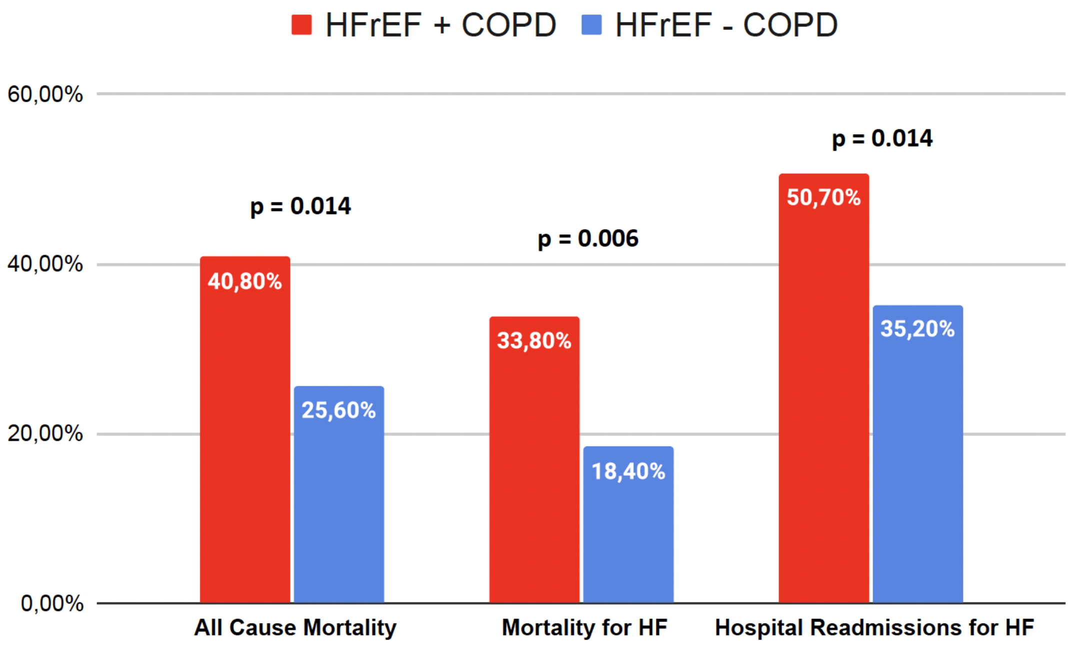

Proportions of patients experiencing all-cause mortality, HF-related mortality, and hospital readmissions for HF during follow-up in the HFrEF + COPD (red bars) and HFrEF - COPD (blue bars) groups. Categorical comparisons between groups were performed using the Pearson chi-squared test. COPD: chronic obstructive pulmonary disease; HF: heart failure; HFrEF: heart failure with reduced ejection fraction.
